# Filarioid infections in wild carnivores: a multispecies survey in Romania

**DOI:** 10.1186/s13071-017-2269-3

**Published:** 2017-07-13

**Authors:** Angela Monica Ionică, Ioana Adriana Matei, Gianluca D’Amico, Jana Ababii, Aikaterini Alexandra Daskalaki, Attila D. Sándor, Dorin Valter Enache, Călin Mircea Gherman, Andrei Daniel Mihalca

**Affiliations:** 1 Department of Parasitology and Parasitic Diseases, Faculty ofVeterinary Medicine, University of Agricultural Sciences and Veterinary Medicine Cluj-Napoca, Calea Mănăștur 3-5, 400372 Cluj-Napoca, Romania; 2 Department of Engineering and Food and Tourism Management, Faculty of Food and Tourism, Transilvania University, Castelului Street, 500014 Braşov, Romania

**Keywords:** Wild carnivores, Dirofilaria spp., Acanthocheilonema reconditum, Infection, Romania

## Abstract

**Background:**

Filarioids are vector-borne parasitic nematodes of vertebrates. In Europe, eight species of filarioids, including zoonotic species, have been reported mainly in domestic dogs, and occasionally in wild carnivores. In Romania, infections with *Dirofilaria* spp. and *Acanthocheilonema reconditum* are endemic in domestic dogs. Despite the abundant populations of wild carnivores in the country, their role in the epidemiology of filarioid parasites remains largely unknown. The aim of the present study was to assess the host range, prevalence and distribution of filarioid infections in wild carnivores present in Romania.

**Methods:**

Between May 2014 and February 2016, 432 spleen samples originating from 14 species of wild carnivores have been tested for the presence of DNA of three species of filarioids (*D. immitis*, *D. repens* and *A. reconditum*).

**Results:**

Overall 14 samples (3.24%) were molecularly positive. The most prevalent species was *D. immitis* (1.62%), accounting for 50% (*n* = 7) of the positive animals. The prevalence of *D. repens* was 1.39%, while that of *A. reconditum* was 0.23%. No co-infections were detected. *Dirofilaria immitis* DNA was detected in five golden jackals, *Canis aureus* (7.58%), one red fox, *Vulpes vulpes* (0.33%), and one wildcat, *Felis silvestris* (10%). The presence of *D. repens* DNA was detected in two red foxes (0.66%), two golden jackals (3.03%), one grey wolf (7.14%), and one least weasel, *Mustela nivalis* (33.33%). *Acanthocheilonema reconditum* DNA was found only in one red fox (0.33%).

**Conclusion:**

The present study provides molecular evidence of filarial infections in wild carnivore species in Romania, suggesting their potential epidemiological role and reports a new host species for *D. repens*.

## Background

Filarioids (Spirurida, Onchocercidae) are vector-borne parasitic nematodes that reside in various tissues of vertebrates [1]. In Europe, eight species of filarioids of four genera (*Dirofilaria*: 2 species; *Acanthocheilonema*: 2 species; *Cercopithifilaria*: 3 species; and *Onchocerca lupi*) have been reported in domestic dogs. Among these, most of the research has been focused on the zoonotic species, namely *D. immitis*, which causes a severe and life-threatening cardio-pulmonary disease in dogs [2], *D. repens*, which resides in the subcutaneous tissues of the canine host and is associated with a variety of dermatological conditions [3, 4] and, more recently, *O. lupi*, which is localized in the connective tissue of the sclera or in the retrobulbar regions of the eye of dogs [5]. *Acanthocheilonema* spp. and *Cercopithifilaria* spp. have a less known ecology, as they seem to be non-pathogenic, and have a minimal clinical importance [6]. Globally, there are several records of free-roaming wild carnivores being naturally infected with filarioid helminths that typically parasitize domestic dogs [7–32]. However, in Europe, only a few extensive studies assessing the prevalence, distribution and mainly patency (i.e. presence of circulating microfilariae) of filarial infections in wild carnivores have been published [7–18, 21].

*Dirofilaria* spp. and *A. reconditum* are distributed in dog populations throughout the country [33], while other species (*C. bainae*, *O. lupi*) have been reported only locally [34, 35]. Romania is mostly a rural country, having an extended wildlife-domestic animal interface, which may facilitate the spreading of canine parasites to wild carnivores, which in turn may act as natural reservoirs. The country is characterized by a high diversity of habitats and biodiversity, having a rich wild carnivore fauna comprising 18 species belonging to 5 families: Mustelidae (10 species), Canidae (4 species), Felidae (2 species), Ursidae (1 species) and Phocidae (1 species) [36]. However, despite this large diversity and abundance of wild carnivores present in the country, their role in the epidemiology of filarioid parasites remains unknown. The aim of the present study was to assess the prevalence and distribution of filarioid infections in wild carnivores present in Romania.

## Methods

Between May 2014 and February 2016, a total of 432 spleen samples originating from 14 species of wild carnivores have been tested (Table 1). The animals were legally hunted, road-killed, or found dead due to natural causes at various locations. For each animal, species, sex, estimated age (juvenile or adult, according to dentition) and collection site were recorded. Collection of samples took place either directly on the field (performed by hunters), or during necropsy. All necropsies were performed at the Department of Parasitology and Parasitic Diseases within the University of Agricultural Sciences and Veterinary Medicine of Cluj-Napoca (Romania). When available, the heart and pulmonary arteries were dissected in order to assess the presence of adults of *D. immitis*. Samples were labelled and stored at -20 °C until further processing.

**Table 1:** Animal species examined in the present study and molecular screening results

Family	Species	*n*	*D. immitis*		*D. repens*		*A. reconditum*	
			*n* (%)	95% CI	*n* (%)	95% CI	*n* (%)	95% CI
Canidae	*Vulpes vulpes*	305	1 (0.33)	0.06–1.83	2 (0.66)	0.18–2.36	1 (0.33)	0.06–1.83
	*Canis aureus*	66	5 (7.58)	2.51–16.80	2 (3.03)	0.37–10.52	0	–
	*Canis lupus*	14	0	–	1 (7.14)	0.18–33.87	0	–
Felidae	*Felis silvestris*	10	1 (10)	0.25–44.50	0	–	0	–
	*Lynx lynx*	4	0	–	0	–	0	–
Mustelidae	*Lutra lutra*	7	0	–	0	–	0	–
	*Meles meles*	5	0	–	0	–	0	–
	*Mustela lutreola*	4	0	–	0	–	0	–
	*Mustela putorius*	3	0	–	0	–	0	–
	*Mustela erminea*	3	0	–	0	–	0	–
	*Mustela nivalis*	3	0	–	1 (33.33)	0.84–90.57	0	–
	*Martes foina*	4	0	–	0	–	0	–
	*Martes martes*	1	0	–	0	–	0	–
Ursidae	*Ursus arctos*	3	0	–	0	–	0	–
Total		432	7 (1.62)	0.79–3.31	6 (1.39)	0.64–3.00	1 (0.23)	0.25–44.50

Genomic DNA was extracted individually from up to 20 mg of splenic tissue using a commercial kit (Isolate II Genomic DNA Kit, Bioline, London, UK) according to the manufacturer’s instructions. The detection of filarioid DNA was performed by means of multiplex PCR discriminating three species of filarioids commonly present in Europe (*D. immitis*, *D. repens* and *A. reconditum*), using primers and protocols available in literature [37]. PCR products were visualised under UV light after electrophoresis in a 2% agarose gel stained with RedSafe™ 20,000× Nucleic Acid Staining Solution (Chembio, St Albans, UK). The size of the attained bands was assessed by comparison to a molecular marker (O’GeneRuler™ 100 bp DNA Ladder, Thermo Fisher Scientific Inc., Waltham, MA, USA).

The frequency and prevalence of infection and their 95% confidence intervals (95% CI) were established using EpiInfo™ 7 software (CDC, USA).

## Results

**Fig. 1:**
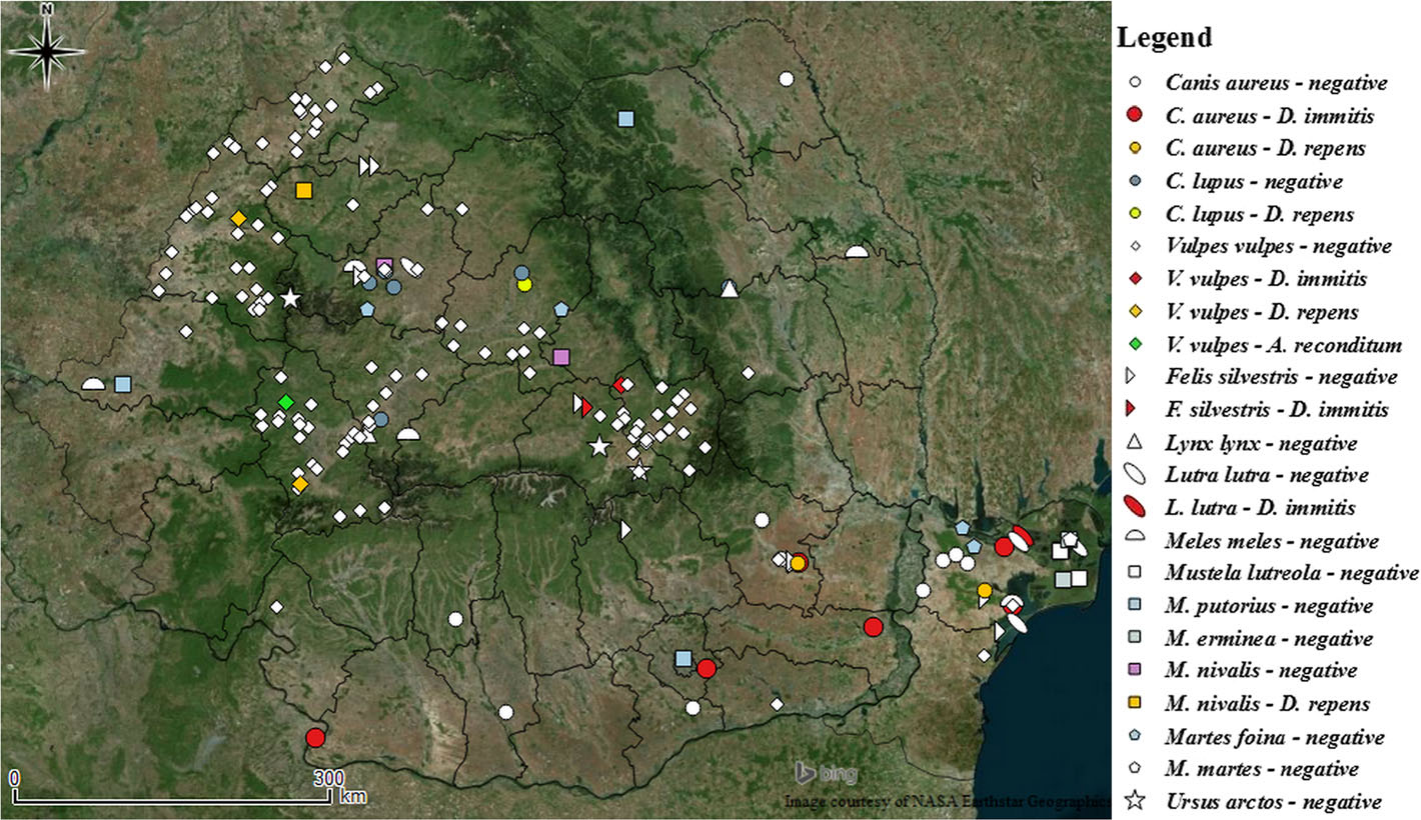
Geographical origin of the samples examined in the country

Overall, 432 spleen samples were tested for the presence of DNA of three filarioid species. A total of 14 samples were positive (3.24%; 95% CI: 1.94–5.37%). *Dirofilaria immitis* DNA was detected in the spleen of five golden jackals, *Canis aureus*, one red fox, *Vulpes vulpes*, and one wildcat*, Felis silvestris* (Table 1). Additionally, heartworms were also present in the right ventricle or pulmonary arteries of one Eurasian otter, *Lutra lutra* (1/7; 16.67%; 95% CI: 0.42–64.12%) and five golden jackals (5/66; 7.58%, 95% CI: 2.51–16.80%). However, all six spleen samples originating from those animals were negative for *D. immitis* DNA (Table 2). The presence of *D. repens* DNA was detected in splenic tissue of two golden jackals, two red foxes, one grey wolf, *C. lupus*, and one least weasel, *Mustela nivalis* (Table 1). *Acanthocheilonema reconditum* DNA was found only in one sample, originating from a red fox (Table 1). No co-infections were detected. The geographical distribution of the positive animals is shown in Fig. 1.

**Table 2:** *Dirofilaria immitis*-positive animals

Host species			Necropsy (*D. immitis*)		PCR result
	Sex	Age	Males	Females	
*Canis aureus*	Female	Adult	2	5	Positive
	Female	Adult	2	3	Positive
	Male	Adult	1	0	Negative
	Female	Juvenile	1	1	Negative
	Female	Adult	2	0	Negative
	Female	Adult	1	2	Positive
	Male	Adult	0	1	Negative
	Male	Adult	1	1	Positive
	Female	Adult	1	3	Positive
	Male	Juvenile	1	2	Negative
*Lutra lutra*	Female	Juvenile	1	2	Negative
*Felis silvestris*	Male	Adult	na		Positive
*Vulpes vulpes*	Female	Adult	na		Positive

*Abbreviation: na* necropsy not performed, spleen sample collected directly in the field

## Discussion

The present study reports the presence of filarioid DNA in spleen samples collected from various species of wild carnivores throughout Romania. For *Dirofilaria* spp. the distribution pattern in wild carnivores in Romania is similar to that recorded in domestic dogs [33, 38–41]. Moreover, most positive animals originated from the south and southeast of the country, where the prevalence of infection in dogs is the highest, with values of up to 26% [33]. As wild carnivores are susceptible hosts, infections occur most often as an epi-phenomenon of dog infection, particularly in overlapping territories [42]. However, infected microfilaremic carnivores may, in the presence of competent vector species, also act as reservoir hosts.

In Europe, *D. immitis* infections have been previously reported from several species of wild carnivores, but patency of infection was rarely evaluated (Table 3). Overall, in the present study, heartworm infections were detected in the case of one Eurasian otter, ten golden jackals, one wild cat and one red fox. However, DNA of *D. immitis* was not detected in all spleen samples, a fact indicating that the molecular positivity may be related to the presence of microfilariae, therefore, the occurrence of a patent infection. The lack of microfilaremia may be related to several factors, including unisexual infections, prepatency, or the hosts’ immune response leading to the clearance of microfilariae [43]. The molecularly negative animals were either harbouring nematodes of the same sex, or were at juvenile age (under one year old) and had died during the winter. This would correspond to a relatively recent (prepatent) infection, considering that the prepatency period ranges between six and nine months [44]. This represents the second record of *D. immitis* infection in two European species, otter and wild cat. Among mustelids, the reservoir status has been demonstrated experimentally for the domesticated form of the ferret (*Mustela putorius furo*) [45]. In the case of otters, the present study provides further evidence of the possibility of infection to occur. Apart from a single case, data regarding *D. immitis* infection in wild cats is currently absent, but their situation is probably similar to that of the domestic ones, which play a minimal epidemiological role, because they generally have a low worm burden and display low levels and a short duration of microfilaremia [46]. In red foxes, the recorded prevalence is considerably lower compared with those in neighbouring countries, such as Bulgaria or Hungary (Table 3). However, in most of these studies, the authors only reported the presence of adult nematodes and microfilaremia was not assessed. Similarly, in Italy the prevalence of adult heartworms in red foxes was of 9.56% (*n* = 50), while microfilaremia was recorded in only 0.38% (*n* = 2) of cases, indicating that red foxes may not be suitable reservoir hosts [7]. The low prevalence in our study may further support this theory.

**Table 3:** An overview of diagnosed filarioid infections in wild carnivore species from Europe

Host species	Country	*D. immitis*		*D. repens*		*A. reconditum*		Reference
		Prevalence in % (method)	Patency^a^	Prevalence in % (method)	Patency (%)^a^	Prevalence in % (method)	Patency (%)^a^	
*Vulpes vulpes*	Bulgaria	5.1 (necropsy)	not assessed	–	–	–	–	[9]
		3.0 (necropsy)	not assessed	–	–	–	–	[12]
		25.22 (necropsy)	not assessed	–	–	–	–	[16]
	Serbia	1.55 (necropsy)	not assessed	–	–	–	–	[14]
		–	–	2.77 (necropsy)	not assessed	–	–	[21]
	Hungary	3.7 (necropsy)	0	–	–	–	–	[15]
	Italy	9.56 (necropsy)	0.38	1.14 (smears)	1.14	10.89 (smears)	10.89	[7]
		6.06 (necropsy)	1.51	0.75 (smears, PCR)	0.75	9.09 (smears, PCR)	9.09	[13]
	Spain	12.7 (necropsy)	not assessed	–	–	–	–	[8]
		0.4 (necropsy)	not assessed	–	–	–	–	[11]
	Romania	0.33 (PCR: spleen)	not assessed	0.66 (PCR: spleen)	not assessed	0.33 (PCR: spleen)	not assessed	Present study
*Canis aureus*	Bulgaria	4.4 (necropsy)	not assessed	–	–	–	–	[9]
		8.9 (necropsy)	not assessed	–	–	–	–	[12]
		37.54 (necropsy)	not assessed	–	–	–	–	[16]
	Serbia	7.32 (necropsy)	not assessed	–	–	–	–	[14]
	Hungary	7.4 (necropsy)	0	–	–	–	–	[15]
	Romania	18.52 (necropsy); 9.26 (PCR: blood)	not assessed	1.85 (PCR: blood)	not assessed	0 (PCR: blood)	not assessed	[17]
		15.15 (necropsy); 7.58 (PCR: spleen)	not assessed	3.03 (PCR: spleen)	not assessed	0 (PCR: spleen)	not assessed	Present study
*Canis lupus*	Bulgaria	5.5 (necropsy)	not assessed	–	–	–	–	[9]
	Serbia	1.43 (necropsy)	not assessed	–	–	–	–	[14]
		–	–	1.63 (necropsy)	not assessed	–	–	[21]
	Macedonia	–	–	10.0 (necropsy)	not assessed	–	–	[21]
	Italy	1 case (necropsy)	not assessed	–	–	–	–	[19]
	Spain	2.1 (necropsy)	not assessed	–	–	–	–	[10]
	Romania	0 (PCR: spleen)	not assessed	7.14 (PCR: spleen)	not assessed	0 (PCR: spleen)	not assessed	Present study
*Felis silvestris*	Serbia	7.69 (necropsy)	not assessed	–	–	–	–	[14]
	Romania	10 (PCR: spleen)	not assessed	0 (PCR: spleen)	not assessed	0 (PCR: spleen)	not assessed	Present study
*Lutra lutra*	Portugal	1 case (necropsy)	not assessed	–	–	–	–	[20]
	Romania	16.67 (necropsy); 0 (PCR: spleen)	not assessed	0 (PCR: spleen)	not assessed	0 (PCR: spleen)	not assessed	Present study
*Martes foina*	Slovakia	0 (PCR: spleen)	not assessed	33.3 (PCR: spleen)	not assessed	0 (PCR: spleen)	not assessed	[22]
*Mustela nivalis*	Romania	0 (PCR: spleen)	not assessed	33.3 (PCR: spleen)	not assessed	0 (PCR: spleen)	not assessed	Present study

^a^Assessed by microscopical visualization of microfilariae

So far, European records of *D. repens* in wildlife include only a handful of cases apart from red foxes (Table 3). To our knowledge, we provide the first evidence for a new host species, the least weasel, *Mustela nivalis*. Given that studies on *D. repens* infection in wild carnivores are mostly limited to singular cases, it is difficult to estimate their role in the epidemiology of this parasite. More comprehensive studies were performed on red foxes in Italy and revealed a relatively low prevalence of microfilaremia (Table 3). The prevalence recorded in foxes in the present study (0.66%) has a similar low value. These data may indicate that red foxes are not efficient reservoir hosts.

*Acanthocheilonema reconditum* is a largely neglected and poorly known species of filarioid. Microfilariae develop and become infective in fleas or lice [47] and require proximity between infected and uninfected hosts [48]. So far, in Europe, among wild carnivores, this parasite has been documented only in red foxes from Italy, with relatively high prevalence values, suggesting their reservoir competence [7, 13]. In the present study a single red fox (0.33%) was positive. In Romania, in dogs, this species seems to be adapted to higher altitudes and to have a relatively large distribution area, despite the low prevalence [33]. However, neither data regarding the climatic requirements for development, nor full distribution maps are currently available for this species.

## Conclusion

The present study provides molecular evidence for filarial infections in wild carnivore species present in Romania, suggesting a potential epidemiological role and demonstrates a new host species for *D. repens*.
